# Anti-Inflammatory Effect of Flavonoids from *Brugmansia arborea* L. Flowers

**DOI:** 10.4014/jmb.1907.07058

**Published:** 2020-01-17

**Authors:** Hyoung-Geun Kim, Davin Jang, Young Sung Jung, Hyun-Ji Oh, Seon Min Oh, Yeong-Geun Lee, Se Chan Kang, Dae-Ok Kim, Dae Young Lee, Nam-In Baek

**Affiliations:** 1 Graduate School of Biotechnology and Department of Oriental Medicinal Biotechnology, Kyung Hee University, Yongin 704, Republic of Korea; 2 Department of Food Science and Biotechnology, Kyung Hee University, Yongin 17104, Republic of Korea; 3 Department of Herbal Crop Research, National Institute of Horticultural and Herbal Science, RDA, Eumseong 27709, Republic of Korea

**Keywords:** COX-2, flavonol glycoside, HPLC, nitric oxide, RAW 264.7, iNOS

## Abstract

*Brugmansia arborea* L. (Solanaceae), commonly known as “angel’s trumpet,” is widely grown in North America, Africa, Australia, and Asia. It has been mainly used for ornamental purposes as well as analgesic, anti-rheumatic, vulnerary, decongestant, and anti-spasmodic materials. *B. arborea* is also reported to show anti-cholinergic activity, for which many alkaloids were reported to be principally responsible. However, to the best of our knowledge, a phytochemical study of *B. arborea* flowers has not yet been performed. Four flavonol glycosides (**1–4**) and one dihydroflavanol (**5**) were for the first time isolated from *B. arborea* flowers in this study. The flavonoids showed significant antioxidant capacities, suppressed nitric oxide production in lipopolysaccharide (LPS)-treated RAW 264.7 cells, and reduced inducible nitric oxide synthase (iNOS) and cyclooxygenase (COX-2) protein production increased by LPS treatment. The contents of compounds **1–4** in *n*-BuOH fraction were determined to be 3.8 ± 0.9%, 2.2 ± 0.5%, 20.3 ± 1.1%, and 2.3 ± 0.4%, respectively, and that of compound 5 in EtOAc fraction was determined to be 12.7 ± 0.7%, by HPLC experiment. These results suggest that flavonol glycosides (**1–4**) and dihydroflavanol (**5**) can serve as index components of *B. arborea* flowers in standardizing anti-inflammatory materials.

## Introduction

Macrophages are one of the major cell types that play critical roles in the human immune system. These cells mainly defend the body against external pathogens through phagocytosis and expression of cytokines. During the immune response, nitric oxide (NO) and excessive cytokine production generated by inducible nitric oxide synthase (iNOS) and cyclooxygenase-2 (COX-2) are produced as mediators of inflammatory responses [1, 2]. Inflammatory cytokines produced during inflammation can accelerate the expression of reactive oxygen species (ROS), thereby damaging tissue. In general, these ROS are eliminated by antioxidant enzymes such as superoxide dismutase and antioxidant agents such as glutathione [3]. However, if the inflammation is associated with disease or a problem in metabolism, the ROS may not be effectively removed [3]. As the inflammatory reaction progresses, mass production of ROS can result in fatal damage to surrounding tissues [4, 5]. To prevent such damage, it may be useful to ingest supplementary antioxidants [3]. However, synthetic anti-inflammatory agents have been reported to be highly toxic and carcinogenic and to interfere with metabolic and respiratory activities of cells. Therefore, development of alternative natural anti-inflammatory agents without side effects is necessary. Our previous study revealed that alcohol extracts of *Brugmansia arborea* flowers inhibited the NO production in RAW 264.7 macrophage cell lines as well as effectively scavenged ABTS and DPPH radicals.

*B. arborea* L. (Solanaceae), commonly known as “angel’s trumpet,” is widely distributed in North America, Africa, Australia, and Asia [6]. *B. arborea* flowers are strongly fragrant, trumpet-shaped, and vary in color from white to ivory-white or cream. The plant has been mainly used for ornamental purposes as well as analgesic, anti-rheumatic, vulnerary, decongestant, and anti-spasmodic materials [6]. In ancient Native American culture, *B. arborea* was used as medicine, a hallucinogenic drug for rituals, poison, and for burial ceremonies [6]. *B. arborea* was also reported to show anti-cholinergic activity, for which many alkaloids were reported to be principally responsible [7-9]. However, the principal components manifesting the anti-inflammatory activities of the flowers have not yet been identified. Our research examined the anti-inflammatory constituents in *B. arborea* flowers. Several previous studies reported that flavonoids exhibited inhibitory effects on NO production [20, 22].

In this study, we isolated and identified the anti-inflammatory flavonoids in *B. arborea* flowers. In addition, the isolated flavonoids were measured for radical scavenging ability through ABTS and DPPH radicals as well as for their anti-inflammatory activities in RAW 264.7 macrophage cells. In this study, we also established the quantitative analysis method for the flavonoids isolated from the *B. arborea* flowers, which led to the standardization process being required for the production of anti-inflammatory drugs.

## Materials and Methods

### Plant Materials

*B. arborea* flowers were provided from Pocheon Herb Island, Korea, in June 2018 and were identified by Professor Dae-Keun Kim, Woosuk University, Jeonju, Korea. A voucher specimen (KHU2018-0036) is reserved at the Laboratory of Natural Products Chemistry, Kyung Hee University, Yongin, Korea.

### General Experimental Procedures

Kieselgel 60 (Merck, Germany), Lichroprep RP-18, 40~60 μm,(Merck) and Sephadex LH-20 (Amersham Biosciences, Sweden) were used for column chromatography (CC). Thin-layer chromatography (TLC) was performed using Kieselgel 60 F_254_ and RP-18 F_254_S (Merck) TLC plates, and the compounds were detected using a Spectroline Model ENF-240 C/F (Spectronics Corporation, USA) UV lamp and a 10% H_2_SO_4_ solution. Deuterium solvents were purchased from Merck Co., Ltd. and Sigma Aldrich Co., Ltd.(USA). Nuclear magnetic resonance (NMR) spectra were recorded on a 400 MHz FT-NMR spectrometer (Varian Inova AS-400, USA). IR spectra were obtained from a Perkin Elmer Spectrum One FT-IR spectrometer (UK). Electrospray ionization mass spectrometry (ESI/MS) spectra were recorded on a Waters Acquity SQD (Waters, USA) and fast atom bombardment mass spectrometry (FAB/MS) spectra were recorded on a JEOL JMS-700 (Japan). Melting points were measured using a Fisher-John’s melting point apparatus (Fisher Scientific, USA) with a microscope; the determined values were uncorrected. Formic acid, ascorbic acid, 2,2’-azino-bis(3-ethylbenzothiazoline-6-sulfonic acid) diammonium salt (ABTS), 1,1-diphenyl-2-picrylhydrazyl (DPPH), lipopolysaccharide (LPS), 3-(4,5-dimethylthiazol2-yl)-2,5-diphenyltetrazolium bromide (MTT), dimethyl sulfoxide (DMSO), Griess reagent and phosphate-buffered saline (PBS) were purchased from Sigma Chemical Co., LLC. Dulbecco’s Modified Eagle’s Medium (DMEM) and fetal bovine serum (FBS) were obtained from HyClone (USA). All other chemicals and reagents used in this study were of analytical or HPLC grade.

### Extraction and Isolation

**Fig. 1:**
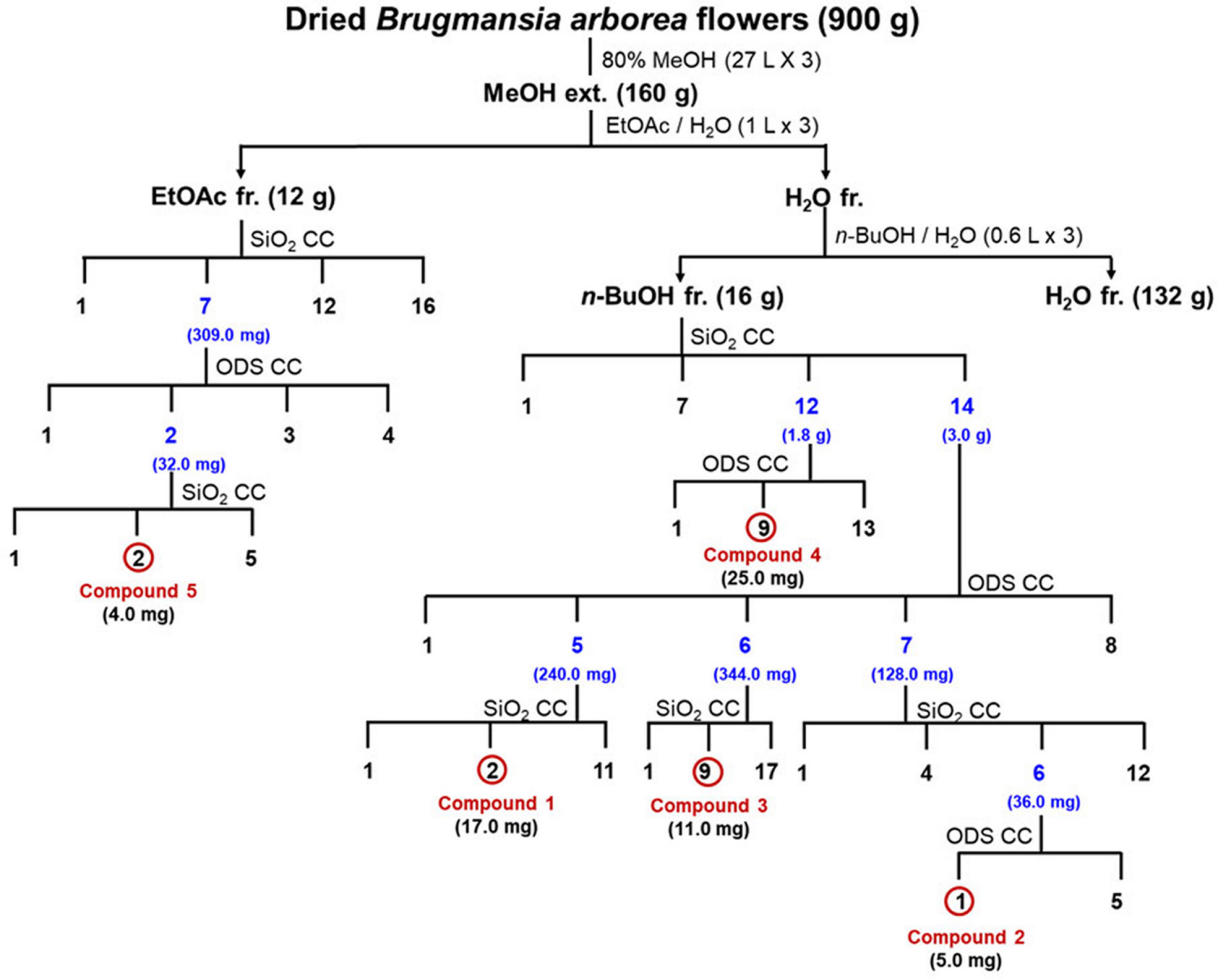
Isolation procedures of compounds **1–5** from *Brugmansia arborea* L. flowers. fr.: fraction; CC: column chromatography; SiO_2_: silica gel; ODS: octadecyl silica gel

**Fig. 2:**
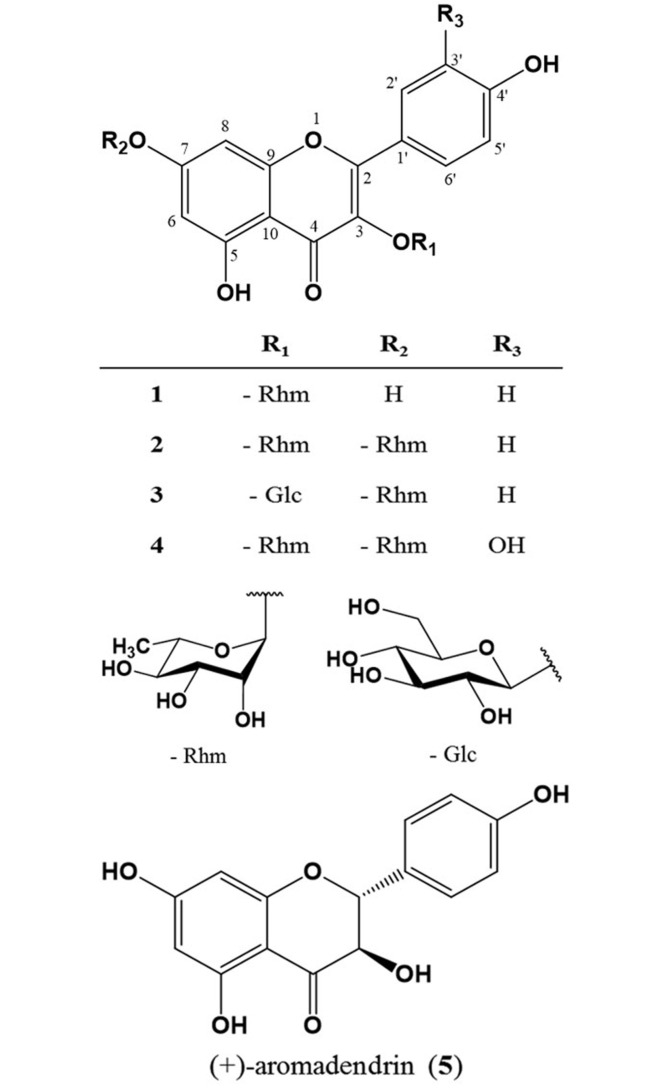
Chemical structures of flavonoids **1−5** isolated from the flowers of *Brugmansia arborea* L. Rhm: *α*-L-rhamnopyranosyl; Glc: β-D-glucopyranosyl

The preparation of methanol extract from *B. arborea* flower (BAF) and solvent fractions, EtOAc (BAFE) and *n*-BuOH (BAFB) fractions, can be referred to in a previous study [10]. The isolation procedures of compounds **1–5** from BAF are presented in Fig. 1 and the chemical structures of compounds **1–5** are presented in Fig. 2.

### Quantitative Analysis of Flavonoids 1-5 Using HPLC Experiment

Quantitative analysis of flavonoids **1–5** in *B. arborea* flowers was performed using HPLC (Alliance e2690; Waters) combined with photodiode array detector (PDA 2998; Waters) and a Poroshell 120 SB-C_18_ (120 Å, 2.7 μm, 4.6 × 150 mm) column (Agilent, USA). The column oven temperature was 30°C, sample injection volume was 5 μl, and detection wavelength was set to 365 nm. Solvent A (H_2_O: formic acid = 99.9: 0.1, v/v) and solvent B (acetonitrile) were used in the mobile phase (0.8 ml/min). The elution program: 92% A/ 8% B at 0 min, 92% A/8% B at 2 min, 88% A/12% B at 3 min, 84%A/16% B at 4 min, 84% A/16% B at 12 min, 80% A/20% B at 15 min, 80% A/20% B at 18 min, 76% A/24% B at 21 min, 70% A/ 30% B at 22 min, 70% A/30% B at 26 min, 50% A/50% B at 28 min, 50% A/50% B at 30 min, 20% A/80% B at 32 min, 20% A/80% B at 33 min, 92% A/8% B at 34 min, and 92% A/8% B at 36 min. For quantitative analysis of **1–5** in *B. arborea* flowers, each compound (1.0 mg) was accurately weighed and dissolved in methanol (1.0 ml) to obtain stock solutions (1.0 mg/ml). Regression curves were developed for each standard with five different concentrations (**1–3**: 500, 250, 125, 50, and 25 μg/ml; **4** and **5**: 250, 125, 50, 25, and 12.5 μg/ml). One milligram obtained from BAFE and BAFB were also accurately weighed and dissolved in 80% aqueous methanol to create stock solutions (1.0 mg/ml). Quantitative analysis was replicated three times.

### Antioxidant Capacities of Solvent Fractions and Flavonoids 1-5

Total antioxidant capacities of solvent fractions and flavonoids **1–5** from BAF were determined using the ABTS and DPPH radical scavenging assays. For the ABTS radical scavenging assay [11], ABTS radical solution was adjusted to the absorbance of 0.650 ± 0.020 at 734 nm. The reaction between ABTS radicals and the appropriately diluted fractions and compounds were allowed at 37°C for 10 min, and then the decrease in absorbance of the resulting solution was measured at 734 nm using a spectrophotometer (SPECTRONIC 200, Thermo Fisher Scientific Inc., Germany). For the DPPH radical assay [12], the absorbance of DPPH radicals in 80% (v/v) aqueous methanol was set to 0.650 ± 0.020 at 517 nm. DPPH radicals and appropriately diluted fractions and compounds were reacted at 23°C for 30 min. The decrease in absorbance of the resulting solution was monitored at 517 nm using a spectro-photometer (SPECTRONIC 200). Antioxidant capacities were expressed as mg vitamin C equivalents (VCE)/g dry weight (DW).

### Cell Culture

The murine macrophage RAW 264.7 cells were obtained from the American Type Culture Collection (ATCC, USA) and cultured in Dulbecco’s modified essential medium supplemented with 10%heat-inactivated fetal bovine serum and 1% penicillin-streptomycin. The cells were incubated in a humidified incubator (BB 15; Thermo Fisher Scientific Inc.) at 37°C in an atmosphere containing 5% CO_2_.

### Cell Viability Assay

RAW 264.7 cells at a density of 4 × 10^4^ cells/well in 96-well plates were pre-cultured for 3 h. After removal of the medium, cells were co-treated with serum-free medium containing 1 μg/ml LPS and the flavonoids **1–5**, BAFE, and BAFB (25, 50, and 100 μg/ml) for 24 h. Then the cells were incubated with 10 μl of 5 mg/ml MTT for 4 h. After the supernatant was removed, 100 μl DMSO was added to each well and shaken for 10 min to dissolve the formazan crystals. Absorbance was measured at 570 nm with a microplate reader instrument (Infinite M200; Tecan Austria GmbH, Austria).

### Determination of Nitrite Production

RAW 264.7 cells at a density of 4 × 10^4^ cells/well in 96-well plates were pre-cultured for 3 hr. After removal of the medium, cells were co-treated with serum-free medium containing 1 μg/ml LPS and the flavonoids **1–5**, BAFE, and BAFB (100 μg/ml) for 24 h. Nitrite accumulation in the culture medium was assessed as an indicator of NO production. Next, 100 μl of the supernatant was transferred to an empty 96-well plate. After adding 100 μl of Griess reagent (1% sulfanilamide and 0.1% naphthylethylenediamine dihydrochloride in 5% phosphoric acid) to each well, absorbance at 540 nm was measured with a microplate reader (Infinite M200; Tecan Austria GmbH). Nitrite concentration was extrapolated from the standard curve of sodium nitrite (0, 2, 4, 8, 16, 32, 64, and 128 μM).

### Western Blot for Protein Measurement

The intracellular content of pro-inflammatory enzymes was measured by western blot. After culturing RAW 264.7 cells for 24 h, they were successively treated using cold PBS and lysis buffer (50 mM Tris-HCl, pH 7.5; 150 mM NaCl; 1 mM EDTA; 20 mM NaF; 0.5% NP-40; and 1% Triton X-100). The supernatant was collected using a centrifuge (15,000 ×g) for 30 min, and protein concentration was measured. The protein quantification method was applied to BCA protein assay. After separation by SDS-PAGE, it was moved to nitrocellulose membrane (0.2 μm), cultured in the primary (iNOS/NOS Type II; BD Biosciences, USA) and secondary antibody (COX-2 Antibody; Cell Signaling Technology, USA) ECL substrate (American Pharmacia Biotech, USA) followed by exposure to film.

### Statistical Analysis

Data are expressed as the mean ± standard deviation for three replicates. Results were assessed using one-way analysis of variance performed using SPSS 23.0 (IBM SPSS Statistics Inc., USA). Tukey-Kramer’s honestly significant difference test was performed to examine significant differences of the means (*p* < 0.05).

## Results and Discussion

### Chemical Structure Elucidation of Flavonoids 1-5

*B. arborea* flowers were extracted using aqueous MeOH, and the concentrates were partitioned into EtOAc, *n*-BuOH, and H_2_O fractions, successively. The EtOAc and *n*-BuOH fractions were used to isolate metabolites and yielded five flavonoids through CC (Fig. 1). All the compounds **1–5** (Fig. 2) showed UV absorption characteristic and exhibited a yellow color on the TLC plate upon spraying with 10% H_2_SO_4_ and heating, suggesting them to be flavonoids. Comparison of NMR (Table 1) and MS data with reported values led to identification of flavonoids **1–5**, kaempferin (**1**) [13, 14], kaempferitrin (**2**) [15], kaempferol 3-*O*-*β*-Dglucopyranosyl- 7-*O*-*α*-L-rhamnopyranoside (**3**) [16], quercetin 3,7-di-*O*-*α*-L-rhamnopyranoside (**4**) [15, 17], and (+)- aromadendrin (**5**) [18]. These compounds were isolated for the first time from BAF in this study.

### Kaempferin (1)

Yellow amorphous powder (CH_3_OH); m.p. 174-175°C; [α]_D_^25^-184° (*c*=0.10, CH_3_OH); positive ESI-MS *m/z* 433 [M + H]^+^; IR (KBr, *v*) 3,400, 1,660, 1,605, 1,500 cm^-1^; ^1^H-NMR (400 MHz, CD_3_OD, δ_H_) and ^13^C-NMR (100 MHz, CD_3_OD, δ_C_) in Table 1.

### Kaempferitrin (2)

Pale yellow powder (CH_3_OH); m.p. 197-198°C; [α]_D_^25^- 231.6° (*c*=0.20, CH_3_OH); positive ESI-MS *m/z* 579 [M + H]^+^; IR (KBr, *v*) 3,369, 1,657, 1,603, 1,493 cm^-1^; ^1^H-NMR (400 MHz, CD_3_OD, δ_H_) and ^13^C-NMR (100 MHz, CD_3_OD, δ_C_) in Table 1.

### Kaempferol-3-*O*-*β*-D-glucopyranosyl-7-*O*-*α*-L-rhamnopyranoside (3)

Pale yellow powder (CH_3_OH); m.p. 261-262°C; [α]_D_^25^- 40.7° (*c*=0.20, CH_3_OH); positive ESI-MS *m/z* 595 [M + H]^+^; IR (KBr, *v*) 3,372, 1,647, 1,600, 1,503 cm^-1^; ^1^H-NMR (400 MHz, CD_3_OD, δ_H_) and ^13^C-NMR (100 MHz, CD_3_OD, δ_C_) in Table 1

### Quercetin-3,7-di-*O*-*α*-L-rhamnopyranoside (4)

Pale yellow powder (CH_3_OH); m.p. 186-187°C; - 55.3° (*c*=0.06, CH_3_OH); positive ESI-MS *m/z* 595 [M + H]^+^; IR (KBr, *v*) 3,371, 1,650, 1,608, 1,503 cm^-1^; ^1^H-NMR (400 MHz, CD_3_OD, δ_H_) and ^13^C-NMR (100 MHz, CD_3_OD, δ_C_) in Table 1.

### (+)-Aromadendrin (5)

Yellow amorphous powder (CH_3_OH); m.p. 59-60°C; [α]_D_^25^+53° (*c*=0.10, CH_3_OH); negative FAB-MS *m/z* 287 [M – H]–; IR (KBr, *v*) 3,530, 1,660, 1,605, 1,550 cm^-1^; ^1^H-NMR (400 MHz, CD_3_OD, δ_H_) and ^13^C-NMR (100 MHz, CD_3_OD, δ_C_) in Table 1.

### Quantitative Analysis of Flavonoids **1–5** in BAFE and BAFB Using HPLC

**Fig. 3:**
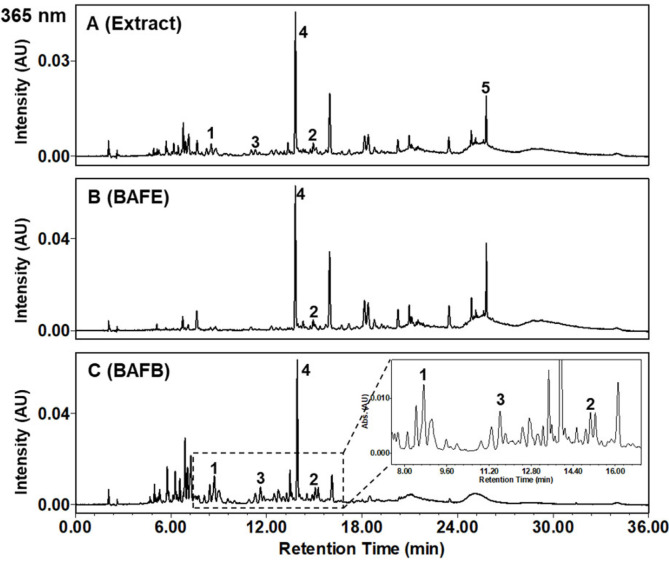
HPLC chromatograms of the *Brugmansia arborea* L. flowers. Extract (**A**), EtOAc fraction (BAFE) (**B**), and *n*-BuOH fraction (BAFB) (**C**) of *B. arborea* flowers.

BAFE and BAFB were quantitatively analyzed by HPLC experiment (Fig. 3). Most peaks were eluted within 36 min, and were detected at 365 nm. Fig. 3B showed compound **5** at 25.8 min and Fig. 3C showed compounds **1–4** at 9.4 min (**1**), 11.3 min (**3**), 13.7 min (**4**), and 15.1 min (**2**), respectively. Each peak was identified to be kaempferin (**1**), kaempferitrin (**2**), kaempferol 3-*O*-*β*-D-glucopyranosyl-7-*O*-*α*-L-rhamnopyranoside (**3**), quercetin 3,7-di-*O*-*α*-L-rhamnopyranoside (**4**), and (+)-aromadendrin (**5**), respectively, by comparing retention times with those of compounds (Fig. 3C). Regression curves were developed for each compound in Table 2. This method was reliable since the r^2^ values of regression curves were all above 0.99. Quantitative analysis was replicated three times. The contents of compounds **1–4** in *n*-BuOH fraction (BAFB) were determined to be 3.8 ± 0.9%, 2.2 ± 0.5%, 20.3 ± 1.1%, and 2.3 ± 0.4%, respectively, and that of compound **5** in EtOAc fraction (BAFE) was determined to be 12.7 ± 0.7%. Flavonoids **3** and **5** were revealed to be major compounds. The aglycone compound, (+)-aromadendrin (**5**), was detected in BAFE, while the glycosides, compounds **1–4** were mainly included in BAFB.

### Radical Scavenging Capacity of Solvent Fractions and Flavonoids 1-5

The antioxidant capacities of solvent fractions and flavonoids **1–5** of *B. arborea* L. flowers by the ABTS and DPPH assays are shown in Table 3. BAFE showed the highest antioxidant capacities in both ABTS and DPPH assays. It was thought that the ethyl acetate fraction mainly contained compounds that contributes more to the antioxidant capacities. The isolated compounds **1–5** showed high ABTS radical scavenging capacities in order 5 > 1 > 4 > 3 > 2. A previous report revealed that the antioxidant capacities measured by ABTS assay depend on the number of -OH groups on flavonoid core [19]. Aromadendrin (**5**) showed the highest antioxidant capacity due to having four -OH groups. The flavonoid monoglycoside, compound **1**, exhibited higher antioxidant capacity than the flavonoid diglycosides, compounds **2–4**, partly due to the steric hindrance caused by the sugar moieties. The aglycone of compound **4**, quercetin, has a catechol structure, which is well known to be the key structure to cause the high radical scavenging activity. As shown in Table 3, the quercetin diglycoside, compound **4**, showed higher antioxidant capacity than the kaempferol diglycosides, compounds **2** and **3** [12, 19].

DPPH radical scavenging activity was almost similar to ABTS radical scavenging activity (Table 3). BAFE showed the highest capacity, and aromadendrin showed higher DPPH scavenging capacity than compounds **1–4** (Table 3). The previous study reported that quercetin-3-rhamnoside (quercitrin) had about half the antioxidant capacity of quercetin [20]. Because the DPPH assay (80% methanol) measures the radical scavenging ability in the non-polar solvent system, compounds **2–4** with two sugar moieties in their structures showed lower DPPH radical scavenging activity than ABTS [11, 21].

### Cell Viability of Solvent Fractions and Flavonoids 1-5

**Fig. 4:**
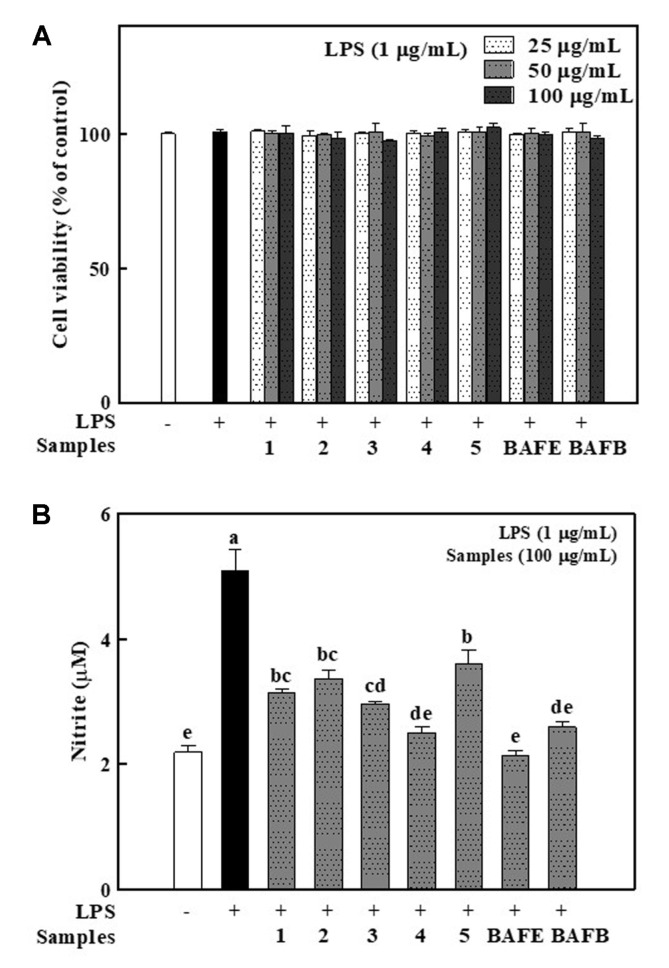
Effects of isolated flavonoids (1-5) and solvent fractions (BAFE and BAFB) on RAW 264.7 cell viability (**A**) and NO production (**B**). Each bar represents the mean ± standard deviation (n = 3). The different letters on the bars indicate significant differences by Tukey- Kramer’s honestly significant difference test (*p* < 0.05). BAFE: *Brugmansia arborea* L. flower EtOAc fraction, BAFB: *Brugmansia arborea* L. flower *n*-BuOH fraction.

The effect of flavonoids upon RAW 264.7 cell viability was measured using MTT assay. All fractions and compounds showed no cytotoxicity at concentrations lower than 100 μg/ml (Fig. 4A). Thus, that concentration was selected for the subsequent experiments.

### Inhibitory Effects of Solvent Fractions and Flavonoids 1-5 on NO Production

BAFE and BAFB fractions and compounds **1–5** were estimated for inhibition strength upon NO production in RAW 264.7 cells provoked by LPS, which was known to be important in inducing inflammation. As shown in Fig. 4B, the treatment of LPS clearly increased the NO production compared to the control. All fractions and compounds suppressed NO production at 100 μg/ml without any apparent toxicity to RAW 264.7 cells. The previous studies also reported that kaempferol glycoside, quercetin glycosides, and aromadendrin suppressed NO production [20, 22, 23]. Compound **4** showed a higher inhibitory effect. Especially, it is remarkable that compound **4** suppressed NO more than compound **2**, due in part to difference of B-ring structure. This can be interpreted that the catechol structure in the flavonoid B ring of quercetin donates electrons, resulting in stable quinones [19]. Catechol moiety in flavonoid plays a critical role in stabilizing the delocalization of electrons around aromatic ring [19]. Removal of ROS using these electrons may lead to inhibition of the inflammatory response, specifically to inhibition of NO formation. It has been reported that flavonoids with 2,3 double bond in C-ring are more effective for inhibition of NO production compared to those without double bond at C-2 and C-3 [24]. Therefore, the aglycone compound, aromadendrin (**5**), suppressed NO production less than the other flavonoids **1–4**. In terms of glycosides, the differences in absorption rate were due to the change in solubility, or steric hindrance that occurred due to the bulky glycosyl group residues can be considered as the cause [25]. This is because the binding of sugars increases the solubility of the flavonoids and the planar structure of the flavonol may be lost [19].

### Inhibitory Effects of Solvent Fractions and Flavonoids 1-5 on Production of iNOS and COX-2

**Fig. 5:**
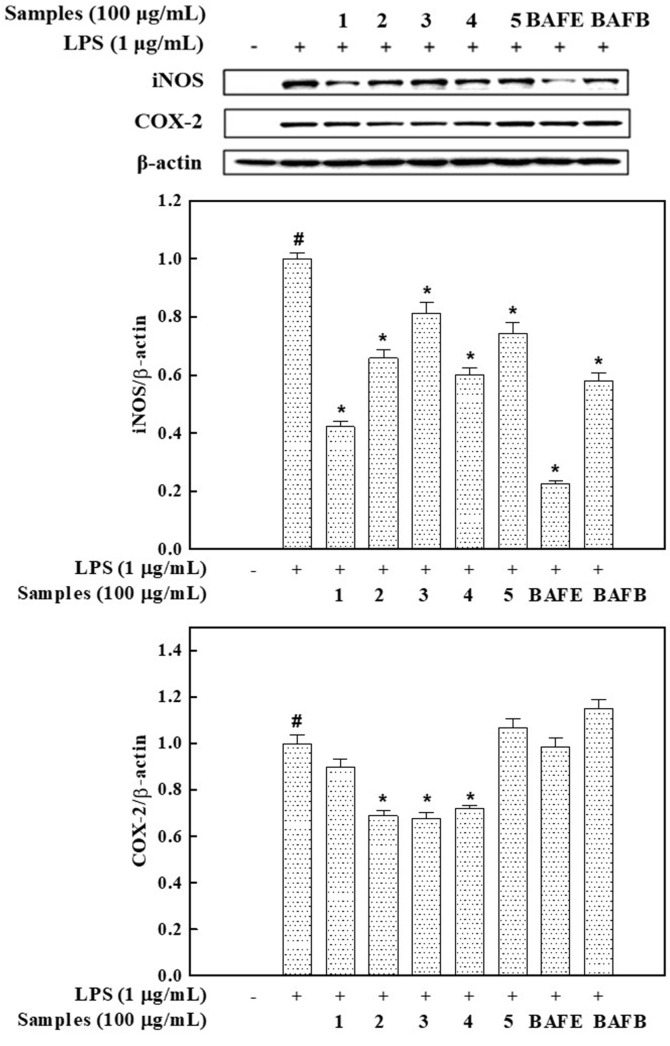
Inhibition effect of compounds **1−5** and solvent fractions from *Brugmansia arborea* L. flower on the protein production of iNOS and COX-2 in RAW 264.7 cells. iNOS and COX-2 protein levels were determined using immunoblotting method. Each bar represents the mean ± S.D. of three independent experiments. *p <0.05 indicates a significant difference from the LPS-treated control.

NO and PGE_2_ production in the inflammatory response are regulated by iNOS and COX-2, respectively [26, 27]. iNOS production is provoked in agreement with typical inflammation stimuli [26]. COX-2 is related to the formation of PGE_2_ from arachidonic acid and is important in regulating the inflammatory response [27]. The inhibition effects on iNOS and COX-2 production were measured using western blot experiments. β-Actin, a housekeeping gene that is expressed at a stable level in varying cellular conditions, was used as a positive control. Fig. 5 showed compounds **1–5** and solvent fractions were found to significantly reduce the iNOS protein expression increased by LPS treatment. This is similar to the results of NO production in Fig. 4. In particular, compound 1 and BAFE extremely decreased production of iNOS by 57.8% and 77.3%, respectively, compared to LPS-treated group. Fig. 5 showed **2–4** also significantly reduced the LPS-induced increase in COX-2 protein production. The previous study proved kaempferol 7-*O*-*β*-D-glucopyranoside contributed to the inhibition of inﬂammatory mediators by suppressing expressions of iNOS and COX-2 at the protein and mRNA level and NF-κB, AP-1, and JAK-STAT pathway in LPS-stimulated RAW 264.7 macrophages [28]. The redox properties of the phenolic compounds can quench NO generated during inflammation. Inhibition of the production of iNOS and COX-2, which produce NO, important mediators of inflammation, implies a decrease in the inflammatory response. The outcome proposes all tested compounds and fractions, especially compound **1** and BAFE, to have high potential as anti-inflammatory agents.

In conclusion, through our research we endeavored to find anti-inflammatory compounds in *B. arborea* L. flowers. Five flavonoids were isolated and identified on the basis of NMR, IR, UV, and MS data. All compounds and solvent fractions were found to inhibit NO and iNOS production in LPS-stimulated RAW 264.7 cells. Especially, compound 1 and BAFE showed high anti-oxidant capacities and were notably effective in inhibiting iNOS protein production. Our data indicate that *B. arborea* L. flower can be useful in the production of antioxidant and anti-inflammatory agents.

## Supplemental Materials



Supplementary data for this paper are available on-line only at http://jmb.or.kr.

## Figures and Tables

**Table 1:** ^1^H-NMR and ^13^C-NMR data of compounds **1–5**. d in ppm, *J* in Hz.

No.	**1** ^a^		**2** ^b^		**3** ^b^		**4** ^a^		**5** ^a^	

	δ_C_	δ_H_ (*J* in Hz)	δ_C_	δ_H_ (*J* in Hz)	δ_C_	δ_H_ (*J* in Hz)	δ_C_	δ_H_ (*J* in Hz)	δ_C_	δ_H_ (*J* in Hz)
**2**	159.8	-	159.8	-	159.8	-	159.8	-	85.5	5.42, d, 11.6
**3**	136.2	-	136.2	-	136.2	-	136.4	-	73.3	4.90, d, 11.6
**4**	180.1	-	180.1	-	180.1	-	179.7	-	183.6	-
**5**	158.0	-	158.0	-	158.0	-	162.9	-	164.9	-
**6**	96.7	6.24, d, 2.4	96.7	6.45, br. s	96.7	6.48, d, 2.4	95.5	6.48, d, 2.0	97.4	6.35, d, 2.0
**7**	165.9	-	165.9	-	165.9	-	163.4	-	168.7	-
**8**	99.8	6.36, d, 2.4	99.8	6.70, br. s	99.8	6.75, d, 2.4	99.8	6.67, d, 2.0	96.2	6.48, d, 2.0
**9**	163.2	-	163.2	-	163.2	-	157.9	-	163.9	-
**10**	105.9	-	105.9	-	105.9	-	107.4	-	101.6	-
**1′**	122.4	-	122.4	-	122.4	-	122.9	-	128.9	-
**2′**	131.9	7.75, d, 8.8	131.9	7.78, d, 8.4	131.9	8.01, d, 8.4	116.9	7.37, d, 2.0	130.3	7.75, d, 8.4
**3′**	116.5	6.97, d, 8.8	116.5	6.92, d, 8.4	116.5	6.98, d, 8.4	146.4	-	116.2	7.23, d, 8.4
**4′**	161.5	-	161.5	-	161.5	-	149.9	-	159.6	-
**5′**	116.5	6.97, d, 8.8	116.5	6.92, d, 8.4	116.5	6.98, d, 8.4	116.3	6.84, d, 8.4	116.2	7.23, d, 8.4
**6′**	131.9	7.75, d, 8.8	131.9	7.78, d, 8.4	131.9	8.01, d, 8.4	122.6	7.30, dd, 8.4, 2.0	130.3	7.75, d, 8.4
**1′′**	103.5	5.34, d, 1.6	103.5	5.37, br. s	101.4	5.50, d, 8.4	103.5	5.37, br. s	-	-
**2′′**	72.1	4.25, dd, 3.2, 1.6	72.0	4.25, br. d, 3.0	74.5	4.25, dd, 8.4, 8.4	72.0	4.25, br. d, 3.2	-	-
**3′′**	72.0	3.75, dd, 9.2, 3.2	71.9	3.75, dd, 9.0, 3.0	77.4	3.78^#^	71.9	3.75, dd, 9.2, 3.2	-	-
**4′′**	73.2	3.34^#^	73.2	3.35^#^	71.0	3.49^#^	73.2	3.33^#^	-	-
**5′′**	71.9	3.33^#^	71.3	3.34^#^	78.0	3.55^#^	71.3	3.32^#^	-	-
**6′′**	17.6	0.98, d, 6.4	17.6	0.99, d, 6.0	62.6	4.55, br. d, 12.0	17.6	0.99, d, 6.0	-	-
						3.87, dd, 12.0, 6.0				
**1′′′**	-	-	101.4	5.53, br. s	103.5	5.51, d, 2.4	100.5	5.53, br. s	-	-
**2′′′**	-	-	72.1	4.30, br. d, 3.0	72.0	4.15, br. d, 3.0	72.1	4.30, br. d, 3.0	-	-
**3′′′**	-	-	71.9	3.90, dd, 9.0, 3.0	71.9	3.78^#^	71.9	3.90, dd, 9.0, 3.0	-	-
**4′′′**	-	-	73.6	3.65^#^	73.2	3.55^#^	73.6	3.65^#^	-	-
**5′′′**	-	-	71.7	3.65^#^	71.3	3.49^#^	71.7	3.65^#^	-	-
**6′′′**	-	-	18.1	1.20, d, 6.0	18.4	1.25, d, 6.0	18.1	1.20, d, 6.4	-	-

^a^^1^H-NMR was measured in CD_3_OD at 400 MHz. ^13^C-NMR was measured in CD_3_OD at 100 MHz.

^b^^1^H-NMR was measured in CD_3_OD at 600 MHz. ^13^C-NMR was measured in CD_3_OD at 150 MHz.

^#^Overlapped

**Table 2:** HPLC data for EtOAc and *n*-BuOH fractions from *Brugmansia arborea* L. flowers.

Com^1^	Frs^2^	RT^3^	Regression curve	R^2^
**1**	*n*-BuOH	11.3	945.27*x*+2856.5	0.999
**2**	*n*-BuOH	16.4	1515.84*x*+10358.4	0.998
**3**	*n*-BuOH	9.5	358.23*x*+1695.4	0.999
**4**	*n*-BuOH	13.5	677.62*x*+1704.2	0.999
**5**	EtOAc	25.8	277.56*x*+2125.6	0.997

^1^Compound

^2^Fraction

^3^Retention time (min)

**Table 3:** Antioxidant capacity of isolated flavonoids and solvent fractions from *Brugmansia arborea* L. flowers.

Sample	Antioxidant capacity (mg vitamin C equivalents/100 mg)	

	ABTS	DPPH
Compound **1**	54.4 ± 1.6^c^^1^	5.2 ± 0.6^d^
Compound **2**	9.9 ± 2.0^e^	1.6 ± 0.2^e^
Compound **3**	15.4 ± 0.7^e^	2.5 ± 0.4^e^
Compound **4**	46.7 ± 2.6^d^	10.6 ± 1.6^c^
Compound **5**	155.6 ± 2.5^b^	22.6 ± 1.1^b^
BAFB^2^	5.6 ± 0.2^c^	2.0 ± 0.2^b^
BAFE^3^	51.3 ± 1.3^a^	13.1 ± 0.4^a^

^1^Data are presented as the mean ± standard deviations (n = 3). Means with different superscripts in the same column are significantly different by Tukey- Kramer’s honestly significant difference test (*p* < 0.05).

^2^*n*-BuOH fraction from *Brugmansia arborea* L. flowers

^3^EtOAc fraction from *Brugmansia arborea* L. flowers
