# Supplementary material for: Anti-Inflammatory Effect of Flavonoids from Brugmansia arborea L. Flowers
Source: J Microbiol Biotechnol. 2020 Jan 17;30(2):163–71. doi: 10.4014/jmb.1907.07058 (PMC9728271; doi:10.4014/jmb.1907.07058)
Supplement: Supplementary file 1 [file JMB-30-2-163-supple.pdf]

J. Microbiol. Biotechnol.
https://doi.org/10.4014/jmb.1907.07058

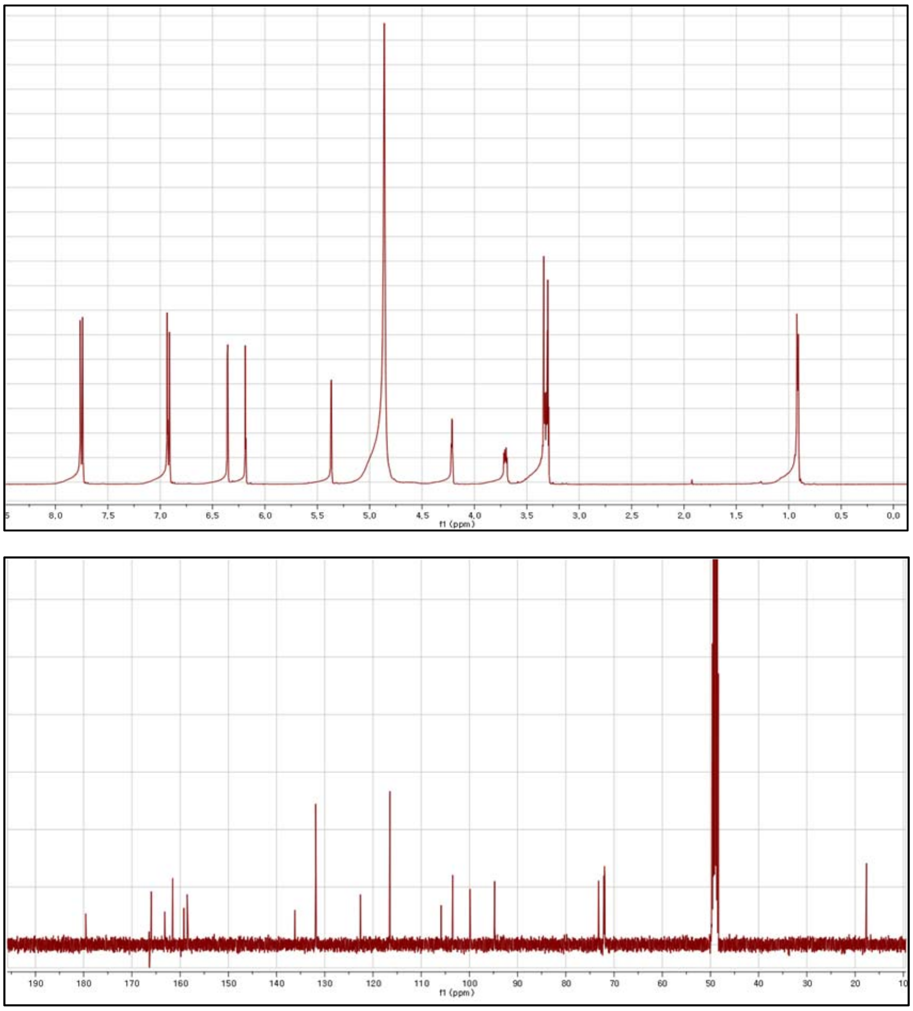

S1. ^{1}H-NMR (400 MHz) and ^{13}C-NMR (100 MHz) spectra of compound **1** (CD_3OD)

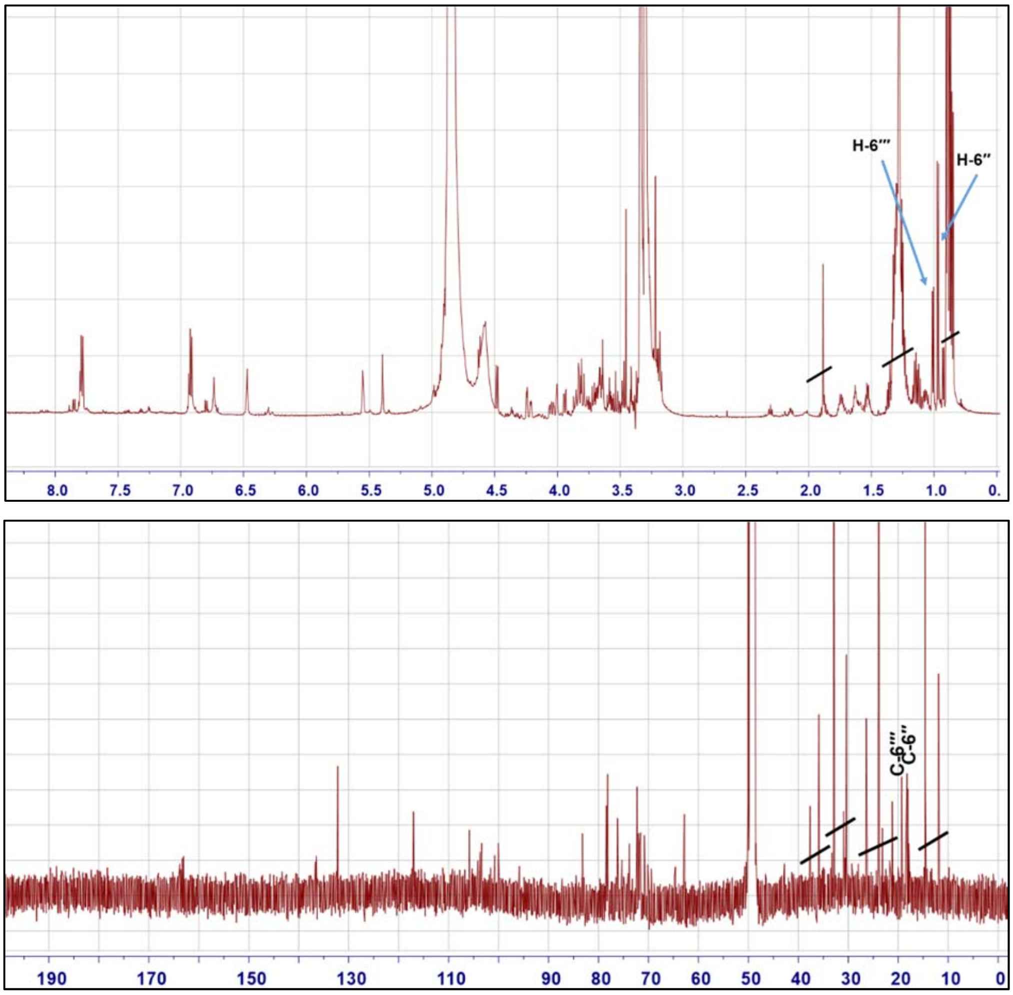

S2. ^{1}H-NMR (600 MHz) and ^{13}C-NMR (150 MHz) spectra of compound **2** (CD_3OD)

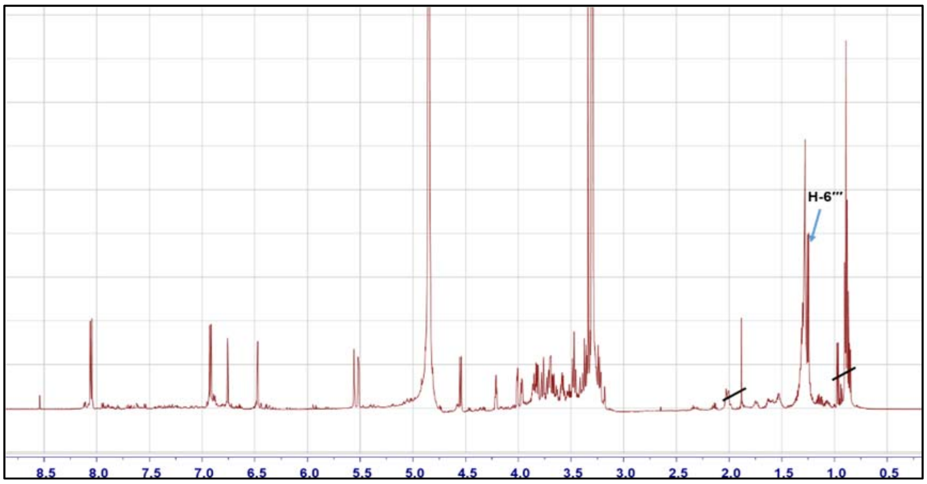

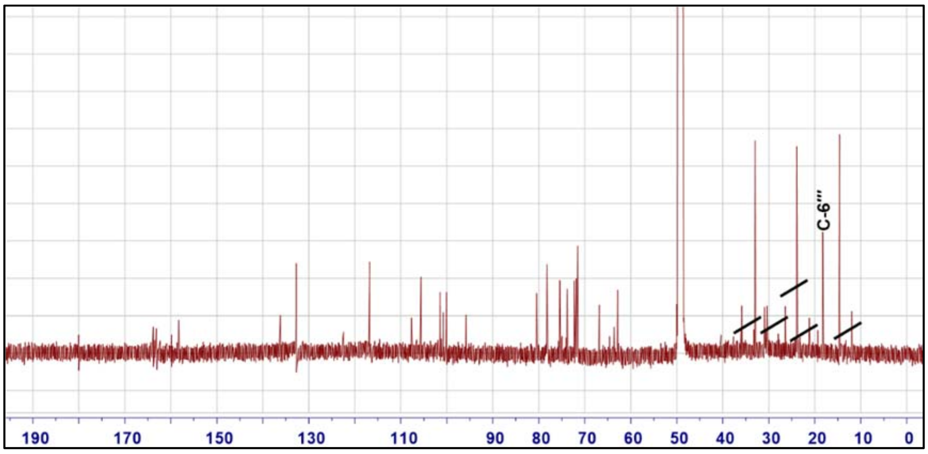

S3. ^{1}H-NMR (600 MHz) and ^{13}C-NMR (150 MHz) spectra of compound **3** (CD_3OD)

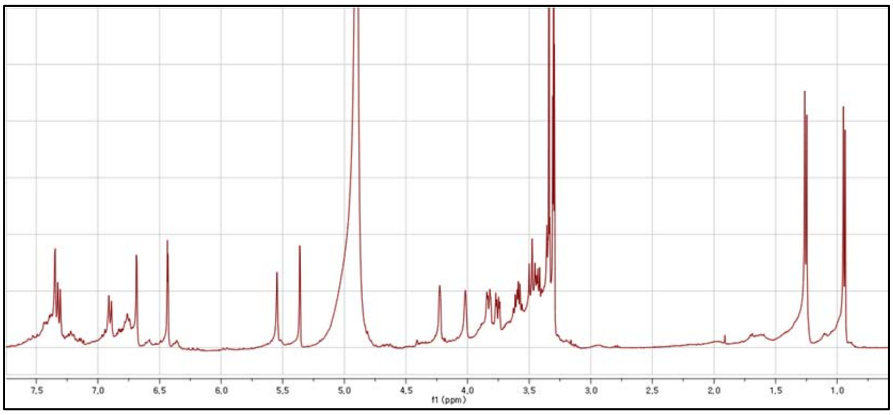

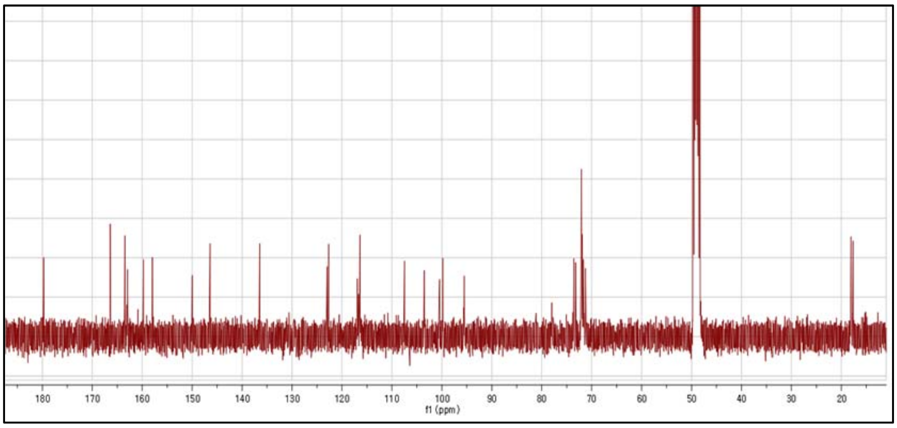

S4. ^{1}H-NMR (400 MHz) and ^{13}C-NMR (100 MHz) spectra of compound **4** (CD_3OD)

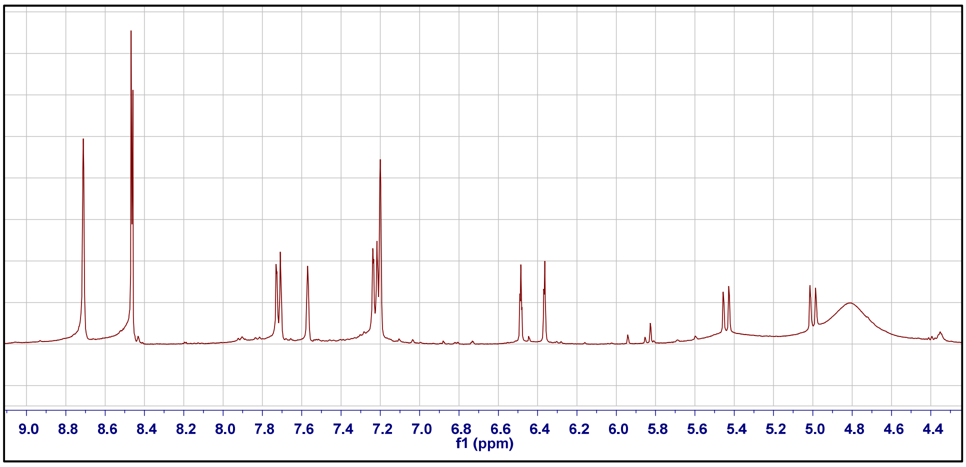

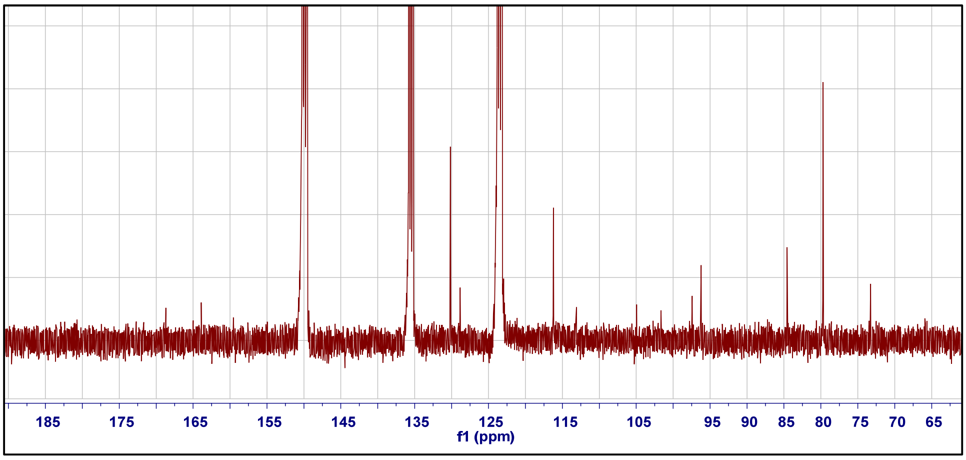

S5. ^{1}H-NMR (400 MHz) and ^{13}C-NMR (100 MHz) spectra of compound **5** (pyridine-d_5)
